# Geospatial analysis and epidemiological aspects of human infections with *Blastocystis hominis* in Mazandaran Province, northern Iran

**DOI:** 10.4178/epih.e2019009

**Published:** 2019-03-28

**Authors:** Shabnam Asfaram, Ahmad Daryani, Shahabeddin Sarvi, Abdol Sattar Pagheh, Seyed Abdollah Hosseini, Reza Saberi, Seyede Mahboobeh Hoseiny, Masoud Soosaraei, Mehdi Sharif

**Affiliations:** 1 Toxoplasmosis Research Center, Mazandaran University of Medical Sciences, Sari, Iran; 2 Student Research Committee, Mazandaran University of Medical Sciences, Sari, Iran; 3 Department of Parasitology, School of Medicine, Mazandaran University of Medical Science, Sari, Iran; 4 Geographic Information System Research Center, Mazandaran University of Medical Science, Sari, Iran

**Keywords:** *Blastocystis hominis*, Geographical information system, Epidemiology, Iran

## Abstract

**OBJECTIVES:**

*Blastocystis hominis* is a very common large intestinal protozoan with global prevalence in humans and non-human hosts. No precise statistics exist regarding the geographical distribution of *Blastocystis* that would enable the identification of high-risk communities. Therefore, the current research aimed to characterize the spatial patterns and demographic factors associated with *B. hominis* occurrence in northern Iran.

**METHODS:**

The current study was performed among 4,788 individuals referred to health centers in Mazandaran Province, from whom stool samples were obtained. Socio-demographic data were gathered using a questionnaire. Samples were examined by a direct wet mount, the formalin-ethyl acetate concentration technique, and trichrome staining. Moran local indicators of spatial association and a geographically weighted regression model were utilized to analyze the results.

**RESULTS:**

Generally, the infection rate of *Blastocystis* parasites was 5.2%, and was considerably higher in the age group of 10-14 years (10.6%) than in other age groups (p=0.005). Our data showed important associations between the occurrence of *B. hominis* and age, residence, job, contact with domestic animals, anti-parasitic drug consumption, and elevation above sea level (p<0.001).

**CONCLUSIONS:**

The current study characterized for the first time the infection rate and risk of *B. hominis* in the north of Iran, and produced a prediction map. It is expected that this map will help policymakers to plan and implement preventive measures in high-risk areas and to manage already-infected patients.

## INTRODUCTION

*Blastocystis hominis* is the most common intestinal protozoan, with a wide geographic distribution that has unclear clinical significance [1]. It is a morphologically variable protozoan that can exist in granular, vacuolar, amoeboid, and cystic forms. Vacuolar forms are most often observed under microscopic examination. The amoeboid forms are considered pathogenic and responsible for the manifestation of clinical symptoms, including various intestinal conditions. With symptoms similar to those of irritable bowel syndrome, transmission of *Blastocystis* occurs by the fecaloral route through the consumption of contaminated water or food [2,3]. Although this parasite can be asymptomatic, in immunocompromised patients it can function as an opportunistic pathogen and cause gastrointestinal disorders [4]. *Blastocystis* spp. are considered to be pathogenic whenever more than 5 parasites are detected in each microscopic field without the presence of other organisms [4,5]. Because of the variable size of the parasite and its similarity to fat drops, yeast, and white blood cells, it has been recommended to apply several diagnostic tests to detect *Blastocystis* in stool specimens [6]. The prevalence of *Blastocystis* infection varies from 1.6% to 16.0% in developed countries, such as Singapore and Japan [7,8] and can reach 60.0% in developing countries including Senegal, Cuba, Brazil, and Argentina [9-12]. In Iran, the total prevalence of *B. hominis* in the total population has been estimated to be 3.0% [13]. Because of the high infection rate, data collection and analysis are essential for identifying high-risk locations, factors related to incidence, and control strategies for *Blastocystis*. The use of a geographic information system is a strategy that could enable a more accurate evaluation of the distribution of the illness in a high-incidence community and improvements in approaches to avoid infection spread. Therefore, using this powerful tool, along with a risk factor questionnaire, constitutes a true environmental health approach [14]. The current study was performed to study the prevalence and geospatial distribution of *Blastocystis* among the total population in Mazandaran Province (in northern Iran) and to identify factors associated with the occurrence of *B. hominis*.

## MATERIALS AND METHODS

### Study area

This cross-sectional study was performed in Mazandaran Province, which is located in northern Iran (35°47ʹ to 36°35ʹN, 50°34ʹ to 54°10ʹE). This province consists of 19 cities and a population of 3,073,943 people. This area has a subtropical climate with an average annual relative humidity of 83%, an average temperature of 18°C, and rainfall occurrence during all four seasons of the year [15].

### Ethics Statement

First, the study protocol was evaluated and approved by the Medical Research Ethics Committee of Mazandaran University of Medical Sciences, Sari, Iran. Informed permission was then obtained from all participants.

### Sample collection

The participants of the current study included 4,788 individuals referred to health centers in Mazandaran Province from January to December 2016. A questionnaire was prepared on the basis of socio-demographic data, and assessed possible parameters related to *Blastocystis* prevalence, including age, sex, site of residence, type of consumed water, job, education, contact with domestic animals, season, and anti-parasitic drug use.

Fresh stool specimens were collected after subjects agreed to participate in the study and completed the questionnaire. The samples were kept in a clean plastic container, fixed in polyvinyl alcohol, and then transferred to the Parasitology Laboratory of Mazandaran University of Medical Sciences.

### Stool examination

All samples were tested with normal saline (0.85% NaCl) for the presence of trophozoites and Lugol iodine staining for the recognition of *Blastocystis* cysts under an optical microscope with × 40 objective magnification. Then, formalin-ether and trichrome staining methods [16] were used to visualize all specimens.

### Geographical data

In our research, data on elevation above sea level (< 500 m, 500- 1,000 m, and > 1,000 m) and distance from the sea (< 10 km, 10- 20 km, and > 20 km) were acquired from Google Earth version 16 (https://www.google.com/earth/). Ecological data (such as temperature, rain, moisture, elevation above sea level, and distance from the sea) were acquired from the Mazandaran Metrological Institute.

### Statistical analysis

The outcomes of the study were analyzed using SPSS version 16.0 (SPSS Inc., Chicago, IL, USA). Local indicators of spatial association were used to characterize the distribution of *B. hominis* and potential risk factors in various areas of the province. Additionally, geographically weighted regression (GWR) was applied to examine the geographical relationships between the occurrence of *Blastocystis* and related main variables, including temperature and precipitation.

## RESULTS

Of the 4,788 individuals referred to health centers, 2,579 (53.9%) were male and 2,209 (46.1%) were female. The average age was 32.39± 17.75 years (range, 1-77 years).

In total, 247 (5.2%) individuals were positive for *B. hominis*. The outcomes demonstrated statistically significant relationships between the prevalence of *B. hominis* and age, occupation, residence, contact with domestic animals, and anti-parasitic drug consumption (p< 0.05) (Table 1).

**Figure 1.**
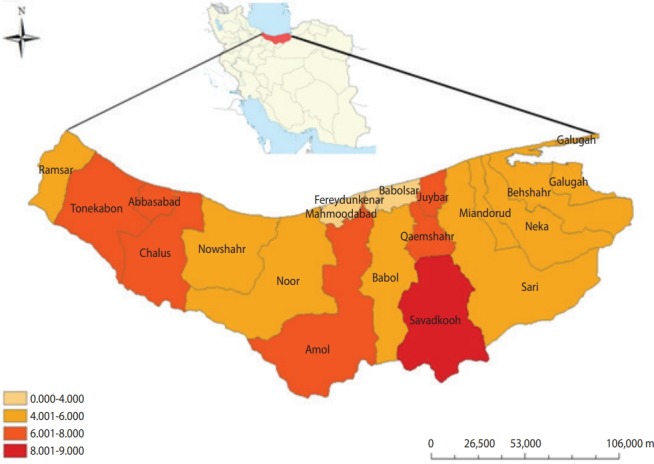
Spatial distribution of *Blastocystis hominis* among the general population in Mazandaran Province. The light-colored areas had the lowest *Blastocystis* rates, while the dark areas had the highest rates by ArcGIS 9.2 (https://support.esri.com/en/).

Based on the outcomes of this research, the prevalence rates of *Blastocystis* according to elevation above sea level was as follows: < 500 m, 4.3% (74 of 1,710); 500-1,000 m, 5.4% (162 of 2,954); and > 1,000 m, 8.9% (11 of 124). Of the meteorological risk factors, only elevation above sea level of > 1,000 m showed a significant difference from sea level (p< 0.05). Mapping the infection rate of *B. hominis* in Mazandaran Province showed that the Savadkooh (8.9%) and Babolsar (1.9%) districts had the maximum and minimum occurrence level of *B. hominis*, respectively (Figure 1).

## DISCUSSION

*Blastocystis* is the most common parasite worldwide and has a global distribution. During recent years, despite improvements in health services, the outcomes of epidemiological studies in numerous parts of the world have indicated that *Blastocystis* infection remains an important health problem in tropical and subtropical areas, particularly in developing countries [17].

Several investigations in Iran have revealed infection rates of 2.4% to 54.5% [18-21]. A meta-analysis in Iran showed an infection rate of 3.0% in the overall population [13]. It seems that differences in the prevalence of *B. hominis* infection may be caused by diverse parameters, such as sample size, type of consumed water, inconsistent laboratory approaches, and ecological parameters.

In the current study, the highest prevalence rate of *B. hominis* was reported in individuals aged 10-14 (10.6%). We observed a significant relationship between *B. hominis* infection and age (p= 0.005), which is in accordance with research performed in Bangladesh [22] and Brazil [23]. Some studies have reported a high prevalence of this infection among all age groups [24,25], possibly due to behavioral patterns and high levels of activity.

The prevalence of *B. hominis* demonstrated significant variation by area (p< 0.001), which is in accordance with studies conducted in South Khorasan of Iran [26] and Turkey [27]. In rural and urban regions, the incidence of *B. hominis* was 7.0% and 3.4%, respectively; the higher rate in rural regions can be explained as the result of poor sanitation, lack of healthy drinking water reservoirs, more contact with the soil, environmental contamination with the cystic form, a large number of households, and geographical factors.

In the current study, 6.2% of the infected subjects had experienced contact with animals (p= 0.001). Several studies have found animal ownership to be a risk factor for *Blastocystis* infection [28- 32].

Our investigation showed that there was a significant relationship between taking anti-parasitic drugs and infection with *B. hominis*. However, 3.2% and 5.7% of subjects with and without a history of anti-parasitic drug consumption were infected with *B. hominis*, respectively (p= 0.001). Similar to several other studies, our findings showed that consumption of anti-parasitic drugs may be an important reason for the reduction in parasitic infections in recent years.

In this study, there was a significant relationship between certain jobs and infection with *B. hominis* (p< 0.001). The prevalence rate of *B. hominis* in farmers (8.7%) was higher than in people with other occupations. Our result is in accordance with the research performed by Banai in Ghazvin Province of Iran [33]. The high prevalence of infection in agriculturists may be because of their high exposure to manure and human excrement in the soil [34].

In this research, similar to other studies, no meaningful relationships were found between the prevalence of *Blastocystis* and sex (p= 0.795), type of consumed water (p= 0.857), education level (p= 0.964), or season (p= 0.399) [35-39].

Despite awareness of the impacts of environmental factors on *B. hominis*, few attempts have been made to map the distribution of this parasite in relation to particular ecological parameters in Iran. Based on the map prepared in this research, Savadkooh district had the maximum prevalence of *B. hominis*. This city is situated in the south of Mazandaran Province, in the northern Alborz Mountains, at a height of 1,000 m. The high rate of *Blastocystis* in Savadkooh district seems to be because of its geographical location, contact with animals, agriculture activities, and the presence of many villages in this region.

Furthermore, the elevation of Savadkooh district above sea level was estimated to be more favorable for cyst persistence [40,41]. The concordance between higher prevalence and elevation could be explained by the fact that cysts are viable for longer in cold climates [41]. Additionally, the transportation of livestock from the plains to mountainous areas in the warm season may influence parasite transmission to different regions. In this study, GWR was applied to examine the geographical relationship of the prevalence of *B. hominis* with several significant factors, including precipitation, temperature, and livestock. The outcomes indicate that 65% and 60% of the prevalence of *Blastocystis* could be explained by contact with domestic animals and rainfall, respectively. This fact highlights the significant impact of these 2 main factors.

Based on our research, the prevalence of *Blastocystis* in mountainous areas may be high because of the more widespread use of unfiltered water sources, high levels of husbandry and agriculture, and lack of good hygiene practices. This geospatial study demonstrated that living in regions with low elevation and converting traditional livestock to industrial livestock could effectively decrease *Blastocystis* infections in different districts in Mazandaran Province. Therefore, the populations living in areas with suitable environmental factors for the parasite are potentially at risk for *Blastocystis* infection.

## Figures and Tables

**Table 1.** Frequency of *Blastocystis hominis* in Mazandaran Province by demographic data and risk factors

Risk factors	Specimens examined	Positive specimens	OR (95% CI)	p-value
Age (yr)				
<5	648	20 (3.0)	1.00 (reference)	
5-9	828	42 (5.0)	0.59 (0.30, 1.00)	0.06
10-14	722	77 (10.6)	3.70 (2.20, 6.50)	0.05
15-24	988	40 (4.0)	1.30 (0.74, 2.40)	0.34
25-39	913	38 (4.1)	1.30 (0.76, 2.40)	0.27
≥ 40	689	30 (4.3)	1.40 (0.77, 2.60)	0.24
Sex				
Male	2,579	135 (5.2)	1.03 (0.79, 1.30)	0.79
Female	2,209	112 (5.0)	1.00 (reference)	
Residence				
Rural	2,273	160 (7.0)	2.10 (1.60, 2.70)	<0.001
Urban	2,515	87 (3.4)	1.00 (reference)	
Consumed water				
Tap	3,984	208 (5.2)	1.00 (reference)	
Well	308	16 (5.1)	1.01 (0.59, 1.80)	0.99
Mineral	496	23 (4.6)	0.88 (0.54, 1.30)	0.66
Job				
Student	1,587	63 (3.9)	1.00 (reference)	
Private business	1,085	58 (5.3)	0.73 (0.49, 1.07)	0.10
Housewife	1,139	56 (4.9)	1.20 (0.84, 1.80)	0.25
Government employee	348	15 (4.3)	1.08 (0.56, 1.90)	0.76
Agriculture	629	55 (8.7)	2.30 (1.50, 3.40)	<0.001
Education				
Illiterate	943	45 (4.7)	1.00 (reference)	
Primary	1,477	70 (4.7)	1.00 (0.67, 1.50)	0.90
High school	1,646	95 (5.7)	1.20 (0.83, 1.80)	0.30
University	722	37 (5.1)	1.07 (0.67, 1.70)	0.80
Contact with domestic animals				
Yes	2,427	151 (6.2)	1.50 (1.10, 2.05)	
No	2,361	96 (4.0)	1.00 (reference)	0.001
Season				
Winter	1,167	60 (5.1)	1.00 (reference)	
Spring	1,196	60 (5.0)	1.02 (0.69, 1.50)	0.92
Summer	1,256	75 (5.9)	1.10 (0.81, 1.60)	0.37
Autumn	1,169	52 (4.4)	0.85 (0.57, 1.20)	0.44
Anti-parasitic drug consumption				
Yes	1,086	35 (3.2)	1.00 (reference)	
No	3,702	212 (5.7)	0.05 (0.36, 0.79)	0.001

Values are presented as number or number (%). OR, odds ratio; CI, confidence interval.
